# Posttraining Alpha Transcranial Alternating Current Stimulation Impairs Motor Consolidation in Elderly People

**DOI:** 10.1155/2019/2689790

**Published:** 2019-07-24

**Authors:** Jost-Julian Rumpf, Alexandru Barbu, Christopher Fricke, Mirko Wegscheider, Joseph Classen

**Affiliations:** Department of Neurology, University of Leipzig, 04103 Leipzig, Germany

## Abstract

The retention of a new sequential motor skill relies on repeated practice and subsequent consolidation in the absence of active skill practice. While the early phase of skill acquisition remains relatively unaffected in older adults, posttraining consolidation appears to be selectively impaired by advancing age. Motor learning is associated with posttraining changes of oscillatory alpha and beta neuronal activities in the motor cortex. However, whether or not these oscillatory dynamics relate to posttraining consolidation and how they relate to the age-specific impairment of motor consolidation in older adults remains elusive. Transcranial alternating current stimulation (tACS) is a noninvasive brain stimulation technique capable of modulating such neuronal oscillations. Here, we examined whether tACS targeting M1 immediately following explicit motor sequence training is capable of modulating motor skill consolidation in older adults. In two sets of double-blind, sham-controlled experiments, tACS targeting left M1 was applied at either 10 Hz (alpha-tACS) or 20 Hz (beta-tACS) immediately after termination of a motor sequence training with the right (dominant) hand. Task performance was retested after an interval of 6 hours to assess consolidation of the training-acquired skill. EEG was recorded over left M1 to be able to detect local after-effects on oscillatory activity induced by tACS. Relative to the sham intervention, consolidation was selectively disrupted by posttraining alpha-tACS of M1, while posttraining beta-tACS of M1 had no effect on delayed retest performance compared to the sham intervention. No significant postinterventional changes of oscillatory activity in M1 were detected following alpha-tACS or beta-tACS. Our findings point to a frequency-specific interaction of tACS with posttraining motor memory processing and may suggest an inhibitory role of immediate posttraining alpha oscillations in M1 with respect to motor consolidation in healthy older adults.

## 1. Introduction

The capacity to acquire new motor skills is an important prerequisite to preserve functional independence across the lifespan. The formation of a new motor skill by integrating different items of a movement into a coherent unit is referred to as motor sequence learning, an ecologically valid and extensively used paradigm to investigate motor learning. In general, formation of a novel sequential motor skill relies on repeated practice and evolves across different phases that are believed to be sustained by distinct mechanisms [1]. Starting with an initial learning phase, skill performance improves “online” across repeated skill execution leading to the formation of an early motor representation (see [2, 3] for review). Immediately subsequent to termination of the online acquisition phase, i.e., “offline,” this initially unstable motor engram is transformed into a more resilient representation in the absence of further skill execution between training sessions. This process, which is referred to as consolidation, may result in offline stabilization or even offline improvements (i.e., offline learning) of skill performance at delayed retesting. The consolidation phase is vulnerable toward interference which may become evident as compromised performance in retention tests (e.g., [4–7]).

Interestingly, while the online acquisition phase of motor sequence learning seems to be relatively unaffected by advancing age, older adults appear to share a specific deficit of the posttraining motor consolidation process [8–12]. A better understanding of the mechanisms underlying this age-related deficit may help to develop strategies restoring motor consolidation in the elderly. Recent studies suggest that age-dependent functional decline may be partially compensated by use of noninvasive brain stimulation [13–15].

With respect to motor consolidation, a large body of research demonstrated an essential role of the primary motor cortex (M1) for consolidation following a training period [3]. The evidence includes the finding that inhibitory repetitive transcranial magnetic stimulation (rTMS) of M1 after motor training blocked consolidation [6, 7]. Furthermore, enhancing excitability of M1 by remote high-frequency rTMS [16] or application of anodal transcranial direct current stimulation (tDCS) [17, 18] immediately after training has been demonstrated to facilitate motor consolidation. In line with these observations in young adults, consolidation was also facilitated in elderly people when tDCS was applied to M1 immediately following motor sequence training [14]. This body of evidence may point to a link between immediate posttraining neuronal excitability changes in M1 and successful consolidation of the training-acquired motor engram.

In addition to an effect on posttraining corticospinal excitability in M1 [16], motor sequence training was also demonstrated to induce modulations of oscillatory activity in the motor cortex during and after motor sequence training in healthy young adults [19]. While changes of motor cortical alpha oscillations were associated with cognitive control during the learning process, changes of beta oscillations were correlated with the magnitude and stabilization of training-induced skill formation and were thus regarded as a possible marker of early cortical reorganization [19].

Transcranial alternating current stimulation (tACS) is a noninvasive brain stimulation technique that is capable of interacting with such neuronal oscillatory activity [20]. Its effects are mainly attributed to the alignment of endogenous oscillatory brain activity with the frequency and phase of the applied alternating current during ongoing stimulation, referred to as entrainment [21]. tACS was demonstrated to modulate local motor cortical excitability as well as functional connectivity of long-range connections during stimulation in a frequency-dependent manner [22, 23]. Of note, tACS also induces after-effects attributed to entrainment echoes and spike-timing-dependent plasticity [24, 25] that cause sustained local excitability and connectivity changes for at least one hour after stimulation [26, 27]. In young adults, application of 20 Hz tACS to the motor cortex concurrent with motor training was shown to facilitate “online” performance increments across the training session [28]. Moreover, beta-band tACS over the motor cortex was demonstrated to also act beneficially with respect to early retrieval of training-acquired motor skill when applied “offline” after training [29].

It is an open question, whether tACS is capable of facilitating consolidation in healthy older adults. Malleability of posttraining consolidation by use of tACS might be a useful tool to compensate age-related deficits of motor consolidation. In the current study, we specifically aimed to investigate whether application of alpha- and/or beta-tACS directed to M1 “offline” after training has an effect on motor consolidation in healthy older adults. We also examined how behavioral effects relate to effects on local oscillatory activity in M1.

## 2. Materials and Methods

### 2.1. Ethics Statement

The study protocol conformed to the principles of the Declaration of Helsinki and was approved by the local ethics committee of the University of Leipzig (study number: 146/16-ek). All participants gave their written informed consent to participate in the study.

### 2.2. Participants

We recruited 40 right-handed healthy older adults aged between 55 and 75 years who were naive to noninvasive brain stimulation techniques. Handedness was determined by the Edinburgh Handedness Inventory [30]. Participants were screened for signs of depression using the Beck Depression Inventory (BDI) [31]. Participants with a BDI score of >13 were excluded from the study. Further exclusion criteria were the presence of serious medical, neurological, or psychiatric diseases. Professional musicians and typists were also excluded. All participants were naïve to the motor sequence learning task and the purpose of the experiment.

Twenty subjects (9 females, age: 68.2 ± 5.8 years, BDI: 4.7 ± 2.7, mean ± SD) participated in experiment 1 which consisted in delivering either active tACS at 10 Hz (“alpha-tACS”) or sham stimulation after completion of the training. Twenty different subjects (14 females; age: 66.4 ± 6.1 years; BDI: 3.7 ± 2.4) were assigned to experiment 2 which consisted in delivering either tACS at 20 Hz (“beta-tACS”) or sham stimulation after completion of the training. Five participants (two participants in experiment 1 and three participants in experiment 2) were excluded from the analyses for an inability to learn the sequence (i.e., average performance across the first five blocks of the training session exceeded task performance at the end of the training session). Additionally, one participant (experiment 1) had to be excluded for not being able to appropriately perform the task (i.e., production of zero correct sequences during several training blocks), and another participant (experiment 1) was excluded for technical problems leading to fragmentary recording of data. Therefore, datasets of 16 remaining eligible participants in experiment 1 (10 Hz alpha-tACS; 9 females; age: 68.5 ± 5.2; BDI: 4.9 ± 2.5) and of 17 participants in experiment 2 (20 Hz beta-tACS; 14 females; age: 66.8 ± 5.7; BDI: 3.7 ± 2.5) were entered in the final analysis.

### 2.3. Experimental Procedure

All participants took part in two different experimental sessions corresponding to one of two types of tACS intervention, i.e., a sham tACS and an active tACS intervention session. Sessions were separated by at least 7 days and balanced in terms of order. Both sessions encompassed a training on a sequential motor task in the morning (between 9 am and 11 am) immediately followed by 15 minutes of sham or active tACS. Motor performance was retested after an interval of 6 hours to assess consolidation of training-induced task skill. Three-channel EEG (C3, Cz, and C4) was recorded before training and after termination of tACS following task training.

### 2.4. Motor Sequence Learning Task

**Figure 1:**
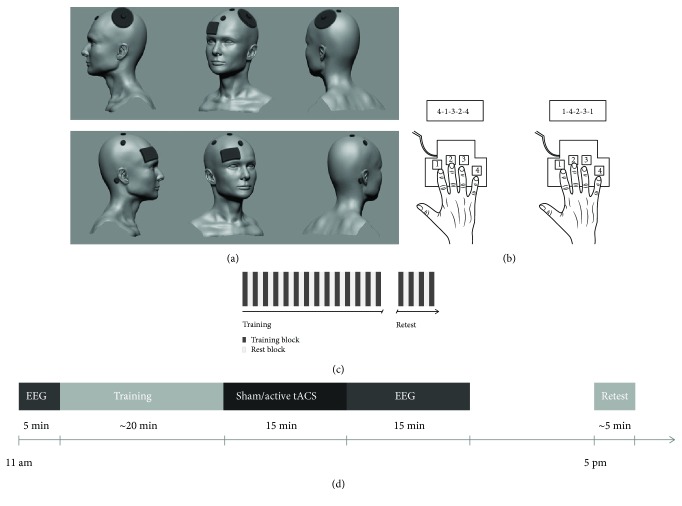
Experimental design. The experiment consisted of two sessions performed on separate days (intersession interval > 7 days). (a) The active EEG electrodes were placed over C3, C4, and Cz positions according to the International 10-20 system. The “donut” tACS electrode was centred around the C3 EEG electrode; the second tACS electrode was placed over the right supraorbital region. (b) Participants performed a different five-item motor sequence in each session with their right hand. (c) The motor sequence training session consisted of 14 practice blocks and the retest of 4 practice blocks, which were separated by 25-second rest blocks. (d) Sham or active tACS was applied over the left primary motor cortex in different sessions. EEG was recorded before training and after stimulation. Consolidation of training-induced speed increments was tested 6 hours later with the trained hand.

Participants were seated in a comfortable chair in front of a computer screen that was used to present task instructions and go/stop signals during performance of the motor task. Motor learning was assessed with a modified version of a well-established explicit sequential finger-tapping task [5]. In each of the two experimental sessions (sham and active tACS session), participants were instructed to practice one of two different, equally difficult finger-tapping sequences with their right hand (sequence 1: “4-1-3-2-4” and sequence 2: “1-4-2-3-1”; 1 = index finger, 2 = middle finger, 3 = ring finger, and 4 = little finger) on a customized keyboard. Sequence 1 and sequence 2 were balanced with respect to assignment to the sham or active session. To verify explicit knowledge of the finger movement sequence, participants were required to slowly repeat the sequence until they were able to correctly repeat it three times in a row. The training phase of each session encompassed 14 successive practice blocks separated by 25-second rest blocks (Figure 1). Participants were instructed to perform the sequence as rapidly as possible while making as few errors as possible. Unbeknownst to the participants, each training block was automatically terminated after 60 key presses to control for the number of finger movements. Thus, a maximum of 12 correct sequence repetitions could be executed within each block of sequence training. The onset of a training block was indicated by a green fixation cross in the middle of the computer screen, which turned red to indicate the onset of a rest block. During rest blocks, participants were instructed to relax their hand until the start of the next practice block. The delayed retest session after an interval of 6 hours to assess offline performance changes consisted of 4 blocks of the task, which were also separated by 25-second rest blocks.

### 2.5. Posttraining Transcranial Alternating Current Stimulation

Posttraining active and sham tACS were delivered (DC-Stimulator Plus, neuroConn, Germany) for 15 minutes immediately after completion of the training session. tACS was applied via a “donut” stimulation electrode (diameter 7.5 cm) with a central recess that was centred around the C3 EEG electrode position (10-20 system) which corresponds to the approximate location of the hand area of M1 [32]. The second stimulation electrode (5 × 7 cm) was placed over the right supraorbital region ipsilateral to the trained hand. Stimulation intensity was increased ramp-like over 8 seconds at the onset of stimulation until a stimulation intensity of 1 mA was reached. In the active tACS condition, stimulation intensity was kept at 1 mA for 15 minutes whereas it slowly faded out after 30 seconds in the sham condition. The fixation of the tACS electrodes to the scalp was assured by Ten20 Conductive Paste (Weaver and Company, Aurora, USA) and by applying close-fitting EEG caps (Easycap, Munich, Germany) over them. The setup of stimulation electrodes was completed together with the EEG setup before the onset of the training part to be able to start stimulation immediately after termination of the training. A separate investigator who was otherwise not involved in the experiment operated the stimulator to ensure that all participants and the experimenter were blind to the type of intervention. At the end of each session, participants were asked to indicate what type of stimulation, sham or active, they believed to have received.

### 2.6. Resting-State EEG Recordings

We recorded resting-state EEG activity to assess changes in peak alpha frequency (PAF) and alpha (8-13 Hz), beta (13-30 Hz), and theta (4-8 Hz) power induced by tACS. EEG was recorded for a period of five minutes prior to motor training (baseline recording) and for 15 minutes immediately after termination of the posttraining tACS intervention (postinterventional recording). During the recording, participants were instructed to keep their eyes open and to fixate on a black cross located about 100 cm in front of them. EEG was recorded from electrodes mounted on the scalp at C3, Cz, and C4, according to the International 10-20 system. An electrode placed on the right mastoid served as reference. Impedances were kept below 5 k*Ω*. EEG signals were amplified using a modified Neuro Prax MR system (neuroConn, Munich, Germany; Ag/AgCl electrodes, Easycap, Munich, Germany; Abralyt HiCl EEG Electrode Gel Easycap, Munich, Germany), recorded fullband (0-1200 Hz), and sampled at 2000 Hz.

### 2.7. Data Analysis and Statistical Methods

#### 2.7.1. Motor Sequence Learning Task

Task execution was recorded with a four-button customized keyboard and processed using customized MATLAB scripts (MathWorks, Natick, USA) to extract speed and accuracy performance. Speed performance was defined as the average time to complete correct sequences within each block (TCS). Accuracy was defined as the ratio of the actual number of correct sequences per block in relation to the maximum number of correct sequences per block (i.e., twelve). Performance development across the training phase was assessed using a repeated measures analysis of variance (rmANOVA) with blocks (14 levels) and type of tACS intervention (two levels: active or sham) as within-subject factors. This allowed to test for performance changes as a function of repeated task training (main effect of block), for differences in the rate of learning with respect to the type of the following tACS intervention (block x intervention interaction), and for overall task performance differences during the training phase (main effect of tACS intervention).

To quantify consolidation, offline posttraining speed and accuracy performance changes were assessed between the “end-of-training performance” (operationally defined as average speed or accuracy performance of the last two blocks of training) and performance in each of the 4 blocks of delayed retesting. rmANOVA of these normalized speed and accuracy measures with blocks (four levels) and type of tACS intervention (two levels) as within-subject factors enabled us to test for overall differences of consolidation between both stimulation sessions (main effect of tACS intervention). rmANOVA also provided information on “online” performance changes across retest blocks driven by additional task training (main effect of block) and on potential differences between both types of tACS intervention with respect to the learning rate across retesting (block x tACS intervention interaction). For all statistical tests, the alpha level was set to 0.05. ANOVAs were checked for violation of sphericity, and *p* values were Greenhouse-Geisser-corrected if necessary. Speed and accuracy measures are reported as the mean with 95% confidence interval (CI). All statistical tests were conducted with SPSS 24 (SPSS, Chicago, IL, USA).

#### 2.7.2. Analysis of EEG Recordings

Processing and analysis of EEG data were performed using MATLAB (MathWorks, Natick, USA) and the FieldTrip toolbox [33]. EEG data was low-pass filtered at 100 Hz and high-pass filtered at 0.5 Hz. Line noise was removed using a bandstop filter at 50 Hz. Datasets were epoched into trials of 10-second length using Hamming windows. Trials containing relevant artefacts were removed manually afterwards. We then calculated peak alpha frequency (PAF) and theta (4-8 Hz), alpha (8-13 Hz), and beta (13-30 Hz) power for the baseline period as well as for seven adjoining two-minute blocks of the postinterventional recording. PAF was calculated by fitting a Gaussian function to the empirical spectra as described by van Albada and Robinson [34]. These parameters were calculated for electrode C3 individually as well as averaged for the three recorded channels (C3, C4, and Cz). Missing values for several single poststimulation blocks due to artefacts were replaced by the average value of the remaining poststimulation blocks. Datasets of participants with no evaluable poststimulation blocks due to artefacts in either of both intervention sessions (three datasets in experiment 1 and five in experiment 2) were removed from further analysis. To detect effects of tACS on poststimulation oscillatory brain activity, we calculated differences of the outlined EEG parameters between each of the seven postinterventional blocks and the baseline measurement before motor training. Differences attributable to tACS were then assessed using repeated measures analysis of variance (rmANOVA) with *time*, corresponding to the two-minute blocks (seven levels), and type of tACS *intervention* (two levels: active or sham) as within-subject factors.

## 3. Results

No side effects, except a slight tingling skin sensation under the electrodes (in 79% of active tACS sessions and 58% of sham tACS sessions) and phosphenes (in 88% of active tACS sessions and 50% of sham tACS sessions), were reported by the participants. Blinding outcome assessment revealed that the rate of correctly identified tACS type (active vs. sham) was 64% across all sessions and did not relevantly differ between experiment 1 (alpha-tACS) and experiment 2 (beta-tACS, *p* = 0.182).

### 3.1. Experiment 1: Posttraining 10 Hz Alpha-tACS

#### 3.1.1. No Difference with respect to Task Learning before Active or Sham Alpha-tACS

**Figure 2:**
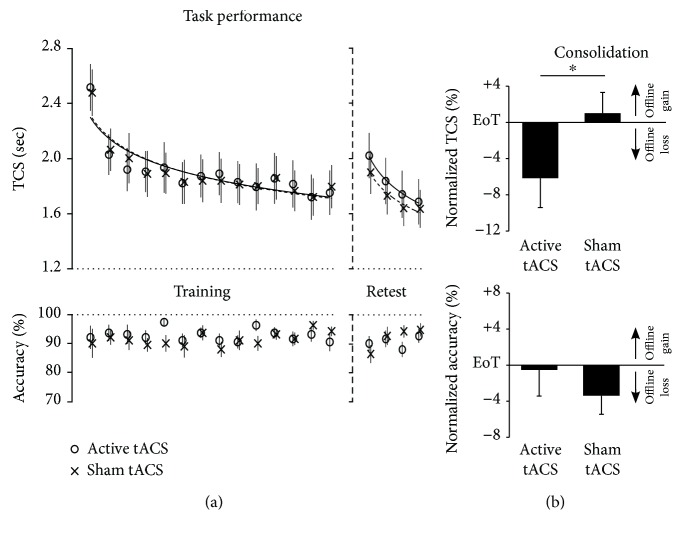
Behavioural results: posttraining active vs. sham 10 Hz (alpha) tACS. (a) Task performance. Mean time to perform a correct sequence per block (TCS) and percentage of correct sequences per block (accuracy) across blocks of training (14 blocks) and delayed retesting (four blocks). Vertical bars represent the standard error of the mean (SEM). (b) Consolidation. Columns represent the mean of normalized speed (TCS) and normalized accuracy performance across the four blocks of delayed retesting, i.e., performance changes relative to the individual “end-of-training performance” (EoT, average PI of last two blocks of training). Positive values indicate offline improvements of speed and accuracy performance (offline gains), while negative values indicate performance decrements (offline loss) relative to EoT. Bars represent SEM. ∗ indicates significant difference of consolidation (*p* < 0.05).

Repeated measures ANOVA with the within-subject factors *intervention* (2 levels) and *block* (14 levels) revealed that participants improved speed performance across the training phase as indexed by decreasing TCS across blocks of training (*F*_(13,195)_ = 30.746, *p* < 0.001; partial eta^2^ = 0.672). Baseline speed performance (TCS of first block of training) was similar during the active (2.52 sec, CI: 2.16–2.88) and the sham intervention (2.48 sec, CI: 2.12–2.84; *p* = 0.765) sessions. Furthermore, there was no significant interaction of *intervention x block* (*F*_(13,195)_ = 0.357, *p* = 0.802; partial eta^2^ = 0.023) indicating similar within-session improvements (i.e., online learning) before posttraining sham and active tACS intervention (Figure 2(a)). Stable asymptotic “end-of-training” speed performance before both sham and active interventions was demonstrated by the finding that there was no significant effect of *block*_B13-B14_ (*F*_(1, 15)_ = 3.001, *p* = 0.104; partial eta^2^ = 0.167) nor a significant interaction of *intervention x block* (*F*_(1, 15)_ = 0.304, *p* = 0.589; partial eta^2^ = 0.020) for the last two blocks of training and was thus considered as the individual baseline against which consolidation effects were assessed. Average “end-of-training” TCS amounted to 1.76 sec (CI: 1.45–2.07) before sham intervention and 1.74 sec (CI: 1.39–2.08; *p* = 0.744) before active intervention.

Accuracy of task performance as indexed by the percentage of correct sequences per block started with an average of 92.3% (CI: 83.9–100.8) in the first block of training in the active intervention session and 90.2% (CI: 79.5–100.8; *p* = 0.369) in the sham intervention session and remained stable across blocks of training before sham and active tACS intervention as rmANOVA revealed no significant effect of *block* (*F*_(13,195)_ = 0.755, *p* = 0.556; partial eta^2^ = 0.048) nor a significant interaction of *block x intervention* (*F*_(13,195)_ = 1.016, *p* = 0.421; partial eta^2^ = 0.063; Figure 2(a)).

#### 3.1.2. Impaired Motor Consolidation following Posttraining 10 Hz Alpha-tACS

Consolidation in terms of speed performance was assessed by normalizing TCS during each block of delayed retesting to the individual “end-of-training performance” (i.e., average TCS of the last two blocks of training). rmANOVA with the within-subject factors *intervention* (2 levels) and *block* (4 levels) applied to these normalized retest TCS values revealed a significant effect of *block* (*F*_(3, 45)_ = 17.636, *p* < 0.001; partial eta^2^ = 0.540) but no significant interaction of *block x intervention* (*F*_(3, 45)_ = 1.280, *p* = 0.294; partial eta^2^ = 0.079) supporting the conclusion that online learning abilities did not differ during delayed retesting following posttraining sham and active tACS intervention. Importantly, rmANOVA showed a significant effect of *intervention* (*F*_(1, 15)_ = 5.238, *p* = 0.037; partial eta^2^ = 0.259) indicating that consolidation was modulated by the type of posttraining tACS intervention. This effect was driven by impaired retest performance when training was followed by 10 Hz alpha-tACS (average normalized retest performance: -6.2%, CI: -13.1–0.7) compared to when training was followed by sham intervention (+1.0%, CI: -3.8–5.8; Figure 2(b)).

Consolidation in terms of accuracy was assessed similarly to speed performance consolidation: percentage of correct sequences per block in each of the retests was normalized to the individual average accuracy performance during the last two blocks of training. rmANOVA conducted on these normalized accuracy values revealed a trend for the factor *block* (*F*_(3, 45)_ = 2.777, *p* = 0.079; partial eta^2^ = 0.156) which was driven by slight improvement of normalized accuracy values across retest blocks. Online learning across blocks of delayed retesting did not differ with respect to posttraining sham and active interventions as no relevant interaction of *block x intervention* (*F*_(3, 45)_ = 1.849, *p* = 0.174; partial eta^2^ = 0.110) was detected. Importantly, rmANOVA revealed no significant effect of *intervention* (*F*_(1, 15)_ = 0.558, *p* = 0.467; partial eta^2^ = 0.036) indicating that impaired speed performance consolidation after posttraining 10 Hz alpha-tACS cannot be explained by an increase in accuracy at the expense of speed performance (i.e., speed accuracy trade-off; Figure 2(b)).

### 3.2. Experiment 2: Posttraining 20 Hz Beta-tACS

#### 3.2.1. No Difference with respect to Task Learning before Sham or Active Beta-tACS

**Figure 3:**
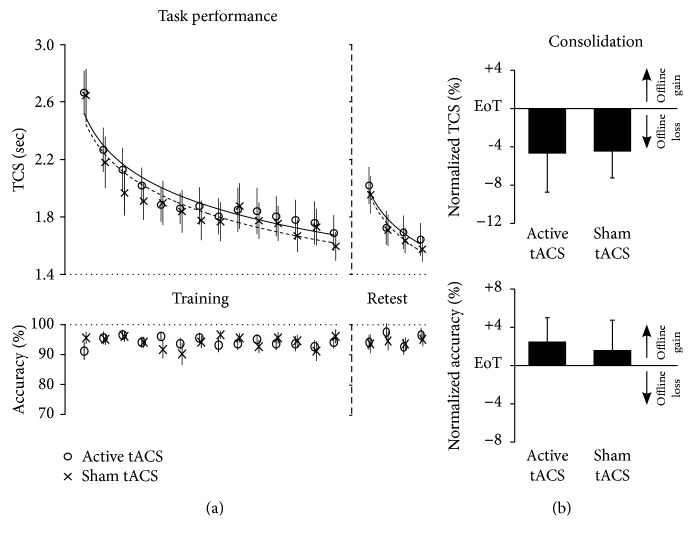
Behavioural results: posttraining active vs. sham 20 Hz (beta) tACS. (a) Task performance. Mean time to perform a correct sequence per block (TCS) and percentage of correct sequences per block (accuracy) across blocks of training (14 blocks) and delayed retesting (four blocks). Vertical bars represent the standard error of the mean (SEM). (b) Consolidation. Columns represent the mean of normalized speed (TCS) and normalized accuracy performance across the four blocks of delayed retesting, i.e., performance changes relative to the individual “end-of-training performance” (EoT, average PI of last two blocks of training). Positive values indicate offline improvements of speed and accuracy performance (offline gains), while negative values indicate performance decrements (offline loss) relative to EoT. Bars represent SEM.

Across blocks of training, rmANOVA showed a significant effect of *block* (*F*_(13,208)_ = 57.214, *p* < 0.001; partial eta^2^ = 0.781) but no significant interaction of *block x intervention* (*F*_(13,208)_ = 0.479, *p* = 0.739; partial eta^2^ = 0.029) demonstrating similar online learning of participants before sham and active tACS intervention comparable to experiment 1. Also comparable to experiment 1, baseline speed performance (TCS in the first block of training) did not differ between the sham (2.65 sec, CI: 2.25–3.04) and active (2.67 sec, CI: 2.34–2.99; *p* = 0.910) intervention session and reached similar “end-of-training” performance (average TCS of last two blocks of training) before sham (1.66 sec, CI: 1.43–1.90) and active intervention (1.72 sec, CI: 1.44–2.01; *p* = 0.390; Figure 3(a)).

Average accuracy in the first block of training amounted to 91.2% (CI: 85.2–97.1) correct sequences in the active intervention session and 95.6% (CI: 91.5–99.6; *p* = 0.208) in the sham intervention session and did not relevantly change across blocks of training before both sham and active tACS interventions as rmANOVA revealed no significant effect of *block* (*F*_(13,208)_ = 0.801, *p* = 0.562; partial eta^2^ = 0.048) nor a significant interaction of *block x intervention* (*F*_(13,208)_ = 0.946, *p* = 0.468; partial eta^2^ = 0.056; Figure 3(a)).

#### 3.2.2. No Effect of Posttraining 20 Hz Beta-tACS on Motor Consolidation

Consolidation in terms of speed and accuracy performance was assessed similarly as described for experiment 1. Repeated measures ANOVA applied to normalized retest TCS values revealed a significant effect of *block* (*F*_(3, 48)_ = 59.186, *p* < 0.001; partial eta^2^ = 0.787) but no significant interaction of *block x intervention* (*F*_(3, 48)_ = 0.384, *p* = 0.695; partial eta^2^ = 0.023) suggesting similar online learning across blocks of delayed retesting after sham and active interventions. The fact that rmANOVA showed no significant effect of *intervention* (*F*_(1, 16)_ = 0.003, *p* = 0.956; partial eta^2^ < 0.001) indicates that speed performance consolidation was not modulated by posttraining beta-tACS compared to the sham intervention. Average normalized TCS after posttraining sham intervention amounted to -4.5% (CI: -10.3–1.2) compared to -4.8% (CI: -13.2–3.7) when training was followed by active beta-tACS (Figure 3(b)).

rmANOVA conducted on normalized retest accuracy revealed no significant effect for the factors *intervention* (*F*_(1, 16)_ = 0.064, *p* = 0.804; partial eta^2^ = 0.004) and *block* (*F*_(3, 48)_ = 1.911, *p* = 0.140; partial eta^2^ = 0.107) nor a significant interaction of *block x intervention* (*F*_(3, 48)_ = 0.437, *p* = 0.727; partial eta^2^ = 0.027) indicating stable accuracy across blocks of retesting after both types of posttraining intervention (i.e., no evidence of speed accuracy trade-off) and no modulation of accuracy performance consolidation by beta-tACS compared to sham intervention (Figure 3(b)).

Of note, to explore the between-group (i.e., between experiments 1 and 2) variation of consolidation in terms of speed following sham tACS, we additionally performed an exploratory between-group comparison of consolidation that revealed no significant difference for average normalized retest TCS with respect to the alpha- vs. beta-tACS sham condition (independent two-sample *t*-test: *t*_(31)_ = 1.564, *p* = 0.128). However, there was also no significant between-group difference of average normalized retest TCS with respect to the corresponding active tACS interventions (*t*_(31)_ = ‐0.274, *p* = 0.784). This indicates that frequency-specific effects of tACS on consolidation can only be inferred from the fact that alpha-tACS impaired consolidation significantly within-group in experiment 1, while beta-tACS induced no effect on consolidation compared to the corresponding sham stimulation in a different group of subjects in experiment 2.

### 3.3. No Effects of Alpha-tACS and Beta-tACS on Postinterventional Resting-State Oscillatory Neuronal Activity in M1

Analysis of the poststimulation normalized spectral power at electrode C3 did not yield a significant main effect of *intervention* following alpha-tACS (experiment 1) for neither PAF (*F*_(1, 12)_ = 3.194, *p* = 0.099; partial eta^2^ = 0.210), alpha power (*F*_(1, 12)_ = 0.676, *p* = 0.427; partial eta^2^ = 0.053), beta power (*F*_(1, 12)_ = 0.498, *p* = 0.494; partial eta^2^ = 0.040), nor theta power (*F*_(1, 12)_ = 0.541, *p* = 0.476; partial eta^2^ = 0.043). For posttraining beta-tACS (experiment 2), we again did not find a significant main effect for *intervention* on beta power (*F*_(1, 11)_ = 0.863, *p* = 0.373; partial eta^2^ = 0.073), PAF (*F*_(1, 11)_ = 1.682, *p* = 0.221; partial eta^2^ = 0.133), alpha power (*F*_(1, 11)_ = 3.983, *p* = 0.071; partial eta^2^ = 0.266), or theta power (*F*_(1, 11)_ = 0.224, *p* = 0.645; partial eta^2^ = 0.020). rmANOVA further revealed no main effect of *time* (*allp* > 0.181) nor an interaction of *time* and *intervention* (*allp* > 0.136). This indicates that neither alpha- nor beta-tACS had a relevant effect on poststimulation PAF, alpha power, beta power, or theta power at the stimulated area compared to the corresponding sham intervention. In addition, besides lacking evidence of poststimulation effects of tACS on oscillatory activity at the stimulated area (C3), no effects on poststimulation PAF, alpha power, beta power, or theta power were detected for the average of oscillatory activity at C3, Cz, and C4 (data not shown). However, we would like to point out that the power of these analyses is limited by the reduced sample size as EEG data of several participants could not be included in the final analysis due to artefacts.

## 4. Discussion

The present study suggests that offline motor memory consolidation following explicit motor sequence training in healthy older adults can be modulated by tACS in a frequency-dependent manner. Importantly, total magnitude and rate of online performance increments across the initial training phase were similar before posttraining application of active and sham 10 Hz (alpha-) tACS as well as before active and sham 20 Hz (beta-) tACS intervention. This indicates that consolidation differences were unlikely to reflect differences of motor engram formation during the online learning phase and suggests that posttraining tACS of M1 is capable of interacting with mechanisms underlying consolidation. Specifically, while posttraining application of beta-tACS of M1 did not modulate consolidation relative to the corresponding sham intervention, consolidation was impaired by posttraining application of alpha-tACS of M1. Importantly, accuracy measures were high from the beginning of the training and revealed no change across the training or across delayed retesting in both experiments. This excludes the fact that changes of speed performance across training and during delayed retesting can be explained by a decrease of accuracy at the expense of speed performance (i.e., speed accuracy trade-off).

Previous studies that employed implicit serial reaction time tasks to investigate motor sequence learning demonstrated selective facilitation of online performance increments in young adults when task execution was combined with concurrent alpha-tACS of M1 [28, 35] as well as when training was combined with concurrent beta-tACS of the motor cortex, while tACS at higher (>20 Hz) or lower (<10 Hz) frequencies had no effect on motor learning [28]. In addition to evidence supporting improved skill acquisition across training (i.e., online learning), beta-tACS directed to the motor cortex during task execution was shown to stabilize training-induced performance increments [28]. Because neurophysiological after-effects of tACS appear to persist for minutes up to at least one hour following termination of stimulation [26, 27, 36], the latter study cannot discriminate whether stabilization of the training-induced motor engram is induced by an interaction of tACS with online processing in M1 during sequence execution or by an interaction with subsequent early offline processing. In young adults, offline application of beta-tACS but not alpha-tACS of M1 immediately after implicit motor sequence training facilitated early retrieval of the trained sequence [29]. Because sequence retrieval was tested immediately following application of tACS and, thus, likely under the influence of tACS after-effects, it was again not possible to discriminate a potential direct interaction of tACS with posttraining consolidation from mere facilitation of performance during early retesting.

In the current study, retesting of sequence performance was performed six hours after the posttraining tACS intervention in order to exclude potential confounds of motor performance during delayed retesting with continued influence of tACS after-effects. While beta-tACS of M1 during the early posttraining period induced no modulation of consolidation in older adults, posttraining alpha-tACS of M1 reduced consolidation compared to the corresponding sham intervention suggesting interference with offline processing of the training-induced motor engram. Because we did not assess sequence performance immediately after the tACS intervention to avoid interference of delayed retesting with tACS after-effects, we remain ignorant about potential transient short-term postinterventional effects of tACS on early retest performance.

How may posttraining tACS of M1 interact with consolidation? Previous studies targeting posttraining motor consolidation in young adults have shown that consolidation after motor learning is disrupted when local excitability in M1 is decreased by low-frequency repetitive transcranial magnetic stimulation [6, 7]. In contrast, consolidation after motor sequence learning appears to be facilitated by immediate posttraining application of noninvasive brain stimulation techniques that are capable of enhancing excitability in M1 such as remote theta-burst stimulation [16] or anodal tDCS [17, 18]. Notably, such facilitatory effects of posttraining noninvasive brain stimulation on consolidation were demonstrated for explicit [16, 18] and for implicit [17] motor learning. In line with these findings in younger adults, consolidation was also enhanced by immediate posttraining application of anodal tDCS of M1 in older adults [14]. Of note, a recent study demonstrated that consolidation in older adults may be more related to the capacity to increase corticospinal excitability in M1 immediately after training than to absolute posttraining corticospinal excitability [8]. This body of evidence points to an important role of posttraining internal or external modulation of excitability in M1 with respect to successful consolidation of training-induced skill formation (cf. [37]). Previous findings suggesting a facilitatory effect on early retrieval by offline beta-tACS of M1 in young adults [29] may be explained by increased cortical excitability in M1 as reported during [22, 38] and after [27, 39] application of beta-tACS of M1. The fact that we did not find a facilitatory effect of posttraining beta-tACS on consolidation in the current study suggests that beta-tACS may less readily enhance local excitability in M1 in older adults. We refrained from testing postinterventional M1 excitability directly to not interfere with consolidation. Because M1 excitability and local beta oscillations are interrelated [40], hints at the presence of tACS-induced modulation of M1 excitability may be derived from analysing postinterventional oscillatory activity in M1. We did not find any relevant difference of local postinterventional beta-band power modulation compared to the corresponding sham intervention. Therefore, we do not have even indirect evidence of tACS-induced alterations of M1 excitability. Because EEG was recorded after, but not during, tACS stimulation, this does not exclude the possibility that tACS may have interacted with M1 oscillations and M1 excitability during ongoing stimulation. Hence, the question why beta-tACS did not alter consolidation must remain open.

Our main finding was that motor consolidation in older adults was impaired by posttraining alpha-tACS compared to the corresponding sham intervention. Several studies suggest an association of alpha oscillations with functional cortical inhibition as alpha amplitude was shown to increase in cortical areas that are not involved in task processing [41–45]. Furthermore, increased alpha power in the M1 region has been shown to be associated with decreased corticospinal excitability [46]. Posttraining entrainment of (inhibitory) alpha oscillations by alpha-tACS may thus impair consolidation by decreasing neuronal excitability in M1 after training. However, in contrast to the effects of application of beta-tACS to M1, several studies found either no change [22, 35, 38, 47] or even enhancement of [48] corticospinal excitability following alpha-tACS of M1. Therefore, the current literature does not support the assumption that posttraining alpha-tACS in older adults may have impaired consolidation by decreasing posttraining corticospinal excitability.

Alternatively, posttraining alpha-tACS may disrupt consolidation independent from any effects on corticospinal excitability. Indeed, Wischnewski and coworkers [27] reported recently that (beta-) tACS-induced changes of corticospinal excitability were not correlated with effects on oscillatory activity. Furthermore, although both the amplitude of motor-evoked potentials and the amplitude of beta oscillations in the motor cortex were each shown to be associated with the level of cortical excitability, they do not seem to mutually correlate strongly [40]. It has been suggested that the EEG alpha rhythm reflects phasic modulations of cortical inhibition [44, 49]. Furthermore, the level of inhibition within the brain influences plasticity induction [50, 51]. Therefore, enhancing oscillatory alpha activity by posttraining alpha-tACS may block consolidation of motor sequence learning, if it involves such mechanisms.

In addition to local processing in M1, online and offline motor sequence learning substantially relies on the dynamic recruitment of a large motor network encompassing M1, parietal cortices, basal ganglia, supplementary motor area, the cerebellum, and hippocampus [52–56]. Recent research demonstrated that storage of sequence-specific information following motor sequence learning relies on the formation of specialized neuronal circuits, which are widely distributed across primary and secondary motor cortices [57]. Furthermore, several studies suggested that synchronization of neuronal activity is central to the induction of plastic network modulation [58, 59]. With respect to the motor system, Stefanou and coworkers [60] recently demonstrated a central role of phase synchronicity of *μ*-rhythm in bilateral sensorimotor cortices for interhemispheric communication. tACS has been demonstrated to be capable of entraining local alpha oscillatory activity [21] as well as long-range connectivity patterns of M1 [23] in young adults. Ageing has been shown to be associated with alterations of functional resting-state connectivity related to learning and early consolidation of a new motor skill [61]. The capacity to induce long-range network plasticity may be specifically challenged in older adults as age-related declines in motor performance have been recently attributed to a breakdown in the functional organization of large-scale brain networks [62]. Thus, one might speculate that older adults may be especially vulnerable to interventions that interfere with communication within the motor consolidation network. Entraining alpha oscillations locally in M1 following training may, thus, alter the physiological temporal dynamics of neuronal oscillations and disturb phase synchronicity between different nodes of the network that is necessary for optimal network communication and successful motor consolidation. However, we are not aware of previous evidence that points to an age-related alteration of functional network interaction specifically at alpha frequency.

As a limitation of our study, exploratory between-group (i.e., between experiments 1 and 2) comparison of consolidation following active tACS revealed no relevant difference with respect to the stimulation frequency. Frequency-specific effects of tACS on consolidation can, thus, only be inferred from the fact that alpha-tACS impaired consolidation significantly within-group, while beta-tACS induced no effect on consolidation compared to the corresponding sham stimulation within a different group of subjects. Of note, despite similar demographic and training characteristics, there is obvious between-group variation of consolidation following sham tACS in experiment 1 (alpha-tACS) and experiment 2 (beta-tACS). However, this between-group difference of the “baseline” capacity to consolidate motor skill acquisition did not reach statistical significance and rather reflects differences of the group-inherent capacity to consolidate motor skill acquisition than suggesting a frequency-specific modulatory effect of sham tACS on consolidation. The interpretation of a frequency-specific effect of tACS on consolidation is further challenged by the fact that we did not detect immediate postinterventional changes of oscillatory brain activity. However, we would like to point out that this does not exclude frequency-specific modulation of neuronal oscillations during tACS application.

## 5. Conclusions

Our results suggest an interaction of posttraining tACS of M1 with offline motor memory consolidation in older adults. While posttraining beta-tACS of M1 did not modulate consolidation relative to the sham condition, the application of alpha-tACS following training significantly disrupted consolidation compared to the corresponding sham intervention. However, we were not able to detect local tACS-induced postinterventional alterations of oscillatory activity in M1. Our findings suggest that posttraining tACS has the capacity to selectively modulate the motor consolidation process. However, further research is needed to elucidate the mechanisms by which tACS interacts with motor memory consolidation.

## Data Availability

The data used to support the findings of this study are available from the corresponding author upon request.
